# The Long-Time Chronoamperometric Current at an Inlaid Microband (or Laminar) Electrode

**DOI:** 10.3390/s130100626

**Published:** 2013-01-04

**Authors:** Christopher G. Bell

**Affiliations:** Mathematical Institute, University of Oxford, 24-29 St Giles', Oxford, 0X1 3LB, UK; E-Mail: bell@maths.ox.ac.uk; Tel.: +44-0-1865-615-131; Fax: +44-0-1865-615-164

**Keywords:** chronoamperometry, two-dimensional, electrode, band, microband, lamina, voltammetry

## Abstract

In this article, we derive an approximate asymptotic analytical expression for the long-time chronoamperometric current response at an inlaid microband (or laminar) electrode. The expression is applicable when the length of the microband is much greater than the width, so that the diffusion of the electrochemical species can be regarded as two-dimensional. We extend the previously known result for the diffusion-limited current response (*Aoki, K. et al. J. Electroanal. Chem. 1987, 225, 19–32* and *Phillips, C.G. J. Electroanal. Chem. 1992, 333, 11*–*32*) to accommodate quasi-reversible reactions and unequal diffusion coefficients of the oxidant and the reductant. Comparison with numerical calculations validates the analytical expression, and we demonstrate that unequal diffusion coefficients can substantially change the current response. Finally, we discuss the form of the long-time current response for a one-step, one-electron redox reaction if the rate constants are modelled in the Butler–Volmer framework, and indicate the importance of choosing the width of the microband appropriately to allow accurate experimental determination of the standard kinetic rate constant and the electron transfer coefficient.

## 1. Introduction

Chronoamperometry is a widely used voltammetric technique [1], whereby the potential at an electrode is initially stepped from a level at which no current is flowing to a level at which the redox reaction at the electrode can progress and a current starts to flow. The current is generated by the exchange of *n* electrons at the electrode surface according to the following redox reaction with forward and backward rate constants, *k̃_f_* and *K̃_b_* (m·s^−1^):

(1) $$\text{Ox}+n\text{e}\overset{{\tilde{k}}_{f}}{\underset{{\tilde{k}}_{b}}{⇌}}\text{Red}$$

**Figure 1.**
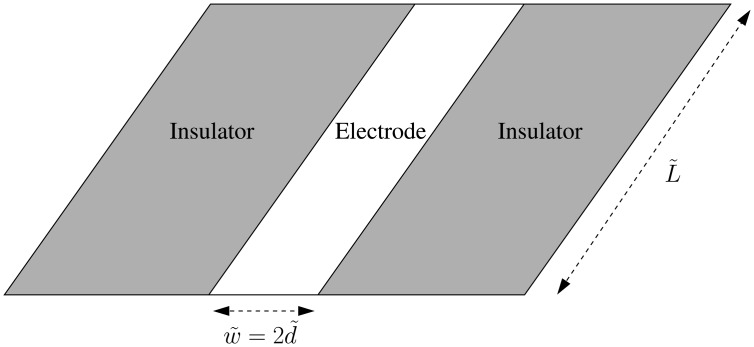
Cartoon of an inlaid microband electrode of width *w̃* = 2*d̃* and length *L̃*. The axial length, *L̃*, of the electrode is much greater than the width, *w̃*, *i.e.*, *L̃* ≫ *w̃*. Then, for time-scales *t̃* such that *t̃* ≪ *L̃*^2^/*D̃*, where *D̃* is the typical size of the diffusion coefficients of the redox species, this allows the two-dimensional-diffusion approximation to be used, since contributions to the current from the three-dimensional diffusion at the ends are negligible.

The information accessible from the chronoamperometric current response can be enhanced by utilising microelectrodes, and in particular ultramicroelectrodes. In addition to reducing the distorting influences of double-layer capacitance and ohmic drop on the Faradaic current [2,3], the small dimensions of the electrodes allow access to information about fast redox kinetics that would previously have been inaccessible [4]. Microelectrodes can be fabricated in many different geometries, such as inlaid disks, inlaid ring-disks and mounted hemispheres, but here we consider an inlaid microband [5,6] such that its length, *L̃*, is much larger than its width, *w̃* = 2*d̃*, as depicted in Figure 1. If the length *L̃* is allowed to be macroscopic, but the width *w̃* is kept microscopic, then the overall current response through the electrode is increased, whilst the advantages of microelectrodes are retained. From a modelling point of view, the advantage of assuming *L̃* ≫ *d̃* is that effects due to the ends of the band can be neglected, and diffusion of the oxidant and the reductant can be regarded as two-dimensional (provided that the time-scales under consideration are such that *t̃* ≪ *L̃*^2^/*D̃*, where *D̃* is the typical size of the diffusion coefficients of the oxidant and the reductant, *D̃_O_* and *D̃_R_*). By considerations of symmetry, the current through an equivalent laminar electrode in free space is twice that of the inlaid band electrode, since it must have identical currents flowing through its upper and lower surfaces.

The majority of the theoretical modelling for the current response at an inlaid microband electrode has involved numerical simulations, using a variety of different techniques [7–21]. The geometry of the electrode makes it more difficult to analyse analytically, due to the discontinuity in the boundary conditions at the edge of the electrode. Oldham [22] accounted for the edge effects to obtain an asymptotic expression for the short-time behaviour in the diffusion-limited regime. Subsequently, Aoki and co-workers developed analytical expressions using the Wiener-Hopf method for the short- [23] and long-time [24] chronoamperometric current responses in the same regime. Phillips and Jansons [25] also derived an analytical expression for the short-time current response using a Brownian motion model for the diffusion of the redox species, and, as part of a more general article on two-and three-dimensional diffusion in microelectrode chronoamperometry, Phillips [26] used matched asymptotic expansions to derive an expression for the long-time current response, which agreed with the first term in the series derived by Aoki *et al.* [24]. In both articles [25] and [26], the inlaid microband is a specific case of more general formulae that can also be applied to many other electrode geometries. Since the short-and long-time asymptotic expressions break down at intermediate times, Szabo *et al.* [27] and Aoki *et al.* [28] have developed approximate analytical expressions to bridge this gap.

All of the expressions derived previously are only valid for the diffusion-limited regime, either due to extreme polarization of the electrode, or when the diffusion coefficients of the oxidant and the reductant are equal, *D̃_O_* = *D̃_R_* = *D̃*, and the following non-dimensional reaction rate (*cf.* [1]) is effectively infinite:

(2) $$\beta =\frac{{\tilde{k}}_{f}\;\tilde{d}}{{\tilde{D}}_{O}}+\frac{{\tilde{k}}_{b}\tilde{d}}{{\tilde{D}}_{R}}$$

**Figure 2.**
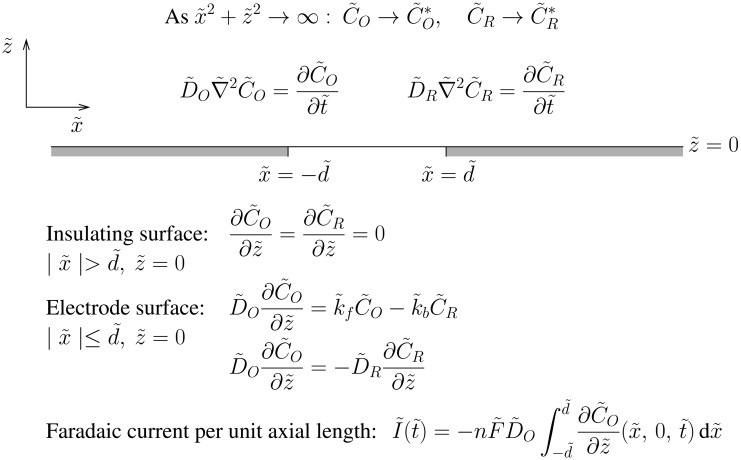
Schematic of the theoretical two-dimensional diffusion problem to be solved for the Faradaic current through an inlaid microband electrode. Two redox species with concentrations *C̃_O_* and *C̃_R_* (mol·m^−3^) diffuse with unequal diffusion coefficients *D̃_O_* and *D̃_R_* (m^2^·s^−1^) above the electrode. Initially the bulk concentrations are uniform everywhere and equal to and; we assume these remain undisturbed as *x̃*^2^ + *z̃*^2^ → ∞. On the surface of the electrode, |*x̃*| ≤ *d̃*, *z̃* = 0, the boundary conditions model the redox reaction in Equation (1) and conservation of matter. The Faradaic current per unit axial length, *Ĩ*(*t̃*) (A·m^−1^), through the electrode is proportional to the integral of the diffusive flux at the electrode surface.

To exploit the capacity of microelectrodes to access information about fast redox kinetics, it is invaluable to understand analytically the effect of the kinetics on the current response. It is also important that the theory accounts for unequal diffusion coefficients of the oxidant and reductant, since this inequality can be significant (see for example [29], where the diffusion coefficient ratio for hexaammineruthenium in different electrolytes is measured to be ≈ 0.71). In this article, we include the effects of finite kinetics and unequal diffusion coefficients, and derive an asymptotic analytical expression for the long-time-dependent Faradaic chronoamperometric current response due to two-dimensional diffusion at an inlaid microband electrode. The solution is valid for time-scales such that *d̃*^2^/*D̃* ≪ *t̃* ≪ *L̃*^2^/*D̃*, where *D̃* is the typical size of the diffusion coefficients of the oxidant and the reductant, *i.e.*, it is valid for time-scales much larger than the diffusive time-scale defined by the width of the electrode, but much shorter than the diffusive time-scale defined by the length of the electrode, when the assumption of two-dimensional diffusion ceases to be valid. For the reader who wishes to skip the mathematical derivation, the final expression for the long-time Faradaic current per unit axial length of the electrode (A·m^−1^) is detailed in Equation (57), and the details of the theoretical problem are displayed in Figure 2. The current depends on a function *Q*(*β*), which is found at each value of the parameter *β* (given by Equation (2)) by solving the integral equation detailed in Equation (41) and substituting the result into Equation (42), or equivalently by solving the boundary value problem given in Equation (70). This function must be found numerically, and we provide a working curve for *Q*(*β*) for 10^−2^ ≤ *β* ≤ 10^3^ in the Supplementary Information. We also derive explicit asymptotic formulae for *Q*(*β*) when *β* is small or large. Comparison with the results of numerical calculations validates the analytical expression for the current response, and we demonstrate that the effect of unequal diffusion coefficients can be significant. Finally, we discuss the implications of using the Butler–Volmer framework [1] to model the rate constants of a one-step, one-electron redox reaction, and indicate that the width of the electrode must be chosen carefully to access accurate information about the standard kinetic rate constant and the electron transfer coefficient from the long-time current response.

## 2. Theory

The theoretical problem is depicted in Figure 2. We consider two species, Ox and Red, diffusing in two-dimensions above an inlaid band electrode with its centre situated at *x̃* = 0, *z̃* = 0 and with width *w̃* = 2*d̃* (m). Throughout the article, tildes indicate dimensional entities. On the surface of the electrode, the species undergo the redox reaction described in Equation (1), and we assume that the potential of the electrode surface is held constant after an initial step, so that *k̃_f_* and *k̃_b_* are both constant. Neglecting any effects due to migration and convection, then the concentrations of Ox and Red, *C̃_O_*(*x̃*, *z̃*, *t̃*) and *C̃_R_*(*x̃*, *z̃*, *t̃*) (mol·m^−3^), satisfy the diffusion equation in *z̃* > 0 with unequal constant diffusion coefficients *D̃_O_* and *D̃_R_* (m^2^·s^−1^). Hence

(3) $$\begin{array}{ccc} {\tilde{D}}_{O}{\tilde{\nabla }}^{2}{\tilde{C}}_{O}=\frac{\partial {\tilde{C}}_{O}}{\partial \tilde{t}}, & {\tilde{D}}_{R}{\tilde{\nabla }}^{2}{\tilde{C}}_{R}=\frac{\partial {\tilde{C}}_{R}}{\partial \tilde{t}}, & \text{in}\;\tilde{z}>0 \end{array}$$

Initially the bulk concentrations of each species are constant everywhere:

(4) $$\begin{array}{ccc} {\tilde{C}}_{O}(\tilde{x},\tilde{z},0)={\tilde{C}}_{O}^{*}, & {\tilde{C}}_{R}(\tilde{x},\tilde{z},0)={\tilde{C}}_{R}^{*}, & \text{in}\;\tilde{z}>0 \end{array}$$

We assume that the bulk concentrations remain undisturbed far away from the electrode as the redox reaction at the electrode progresses, so that the far-field boundary conditions are:

(5) $$\begin{array}{ccc} {\tilde{C}}_{O}\to {\tilde{C}}_{O}^{*}, & {\tilde{C}}_{R}\to {\tilde{C}}_{R}^{*}, & \text{as}\;{\tilde{x}}^{2}+\;{\tilde{z}}^{2}\to \infty \end{array}$$

On the insulating surface, there is no flux of both species:

(6) $$\begin{array}{cc} \frac{\partial {\tilde{C}}_{O}}{\partial \tilde{z}}=\frac{\partial {\tilde{C}}_{R}}{\partial \tilde{z}}=0, & \text{on}|\tilde{x}|>\tilde{d},\tilde{z}=0 \end{array}$$

On the electrode surface, the boundary conditions are determined by the redox reaction in Equation (1) and conservation of matter:

(7) $$\begin{array}{cc} \begin{array}{l} {\tilde{D}}_{O}\frac{\partial {\tilde{C}}_{O}}{\partial \tilde{z}}={\tilde{k}}_{f}\;{\tilde{C}}_{O}-{\tilde{k}}_{b}{\tilde{C}}_{R},\\ {\tilde{D}}_{O}\frac{\partial {\tilde{C}}_{O}}{\partial \tilde{z}}=-{\tilde{D}}_{R}\frac{\partial {\tilde{C}}_{R}}{\partial \tilde{z}}, \end{array}\} & \text{on}|\tilde{x}|\leq \tilde{d},\tilde{z}=0 \end{array}$$

The Faradaic current per unit axial length of the electrode, *Ĩ*(*t̃*) (A·m^−1^), is given by

(8) $$\tilde{I}(\tilde{t})=-n\tilde{F}{\tilde{D}}_{O}\int_{-\tilde{d}}^{\tilde{d}}\frac{\partial {\tilde{C}}_{O}}{\partial \tilde{z}}(\tilde{x},0,\tilde{t})\;d\tilde{x}$$

where *F̃* is Faraday's constant (96,485 C·mol^−1^) and we recall that *n* is the number of electrons transferred in the redox reaction.

To non-dimensionalise the problem, we choose the following scalings, where *d̃* is half the width of the band:

(9a) $$\begin{array}{ccc} \tilde{x}=\tilde{d}x, & \tilde{z}=\tilde{d}z, & \tilde{t}=\frac{{\tilde{d}}^{2}}{\tilde{D}}t \end{array}$$

(9b) $$\begin{array}{cccc} {\tilde{D}}_{O}=\tilde{D}D_{O}, & {\tilde{D}}_{R}=\tilde{D}D_{R} & {\tilde{k}}_{f}=\frac{{\tilde{D}}_{O}}{\tilde{d}}k_{f}, & {\tilde{k}}_{b}=\frac{{\tilde{D}}_{O}}{\tilde{d}}k_{b} \end{array}$$

(9c) $$\begin{array}{cc} {\tilde{C}}_{O}={\tilde{C}}_{O}^{*}-\left(\frac{k_{f}\;{\tilde{C}}_{O}^{*}-k_{b}{\tilde{C}}_{R}^{*}}{\beta }\right)C_{O}, & {\tilde{C}}_{R}={\tilde{C}}_{R}^{*}-\left(\frac{k_{f}\;{\tilde{C}}_{O}^{*}-k_{b}{\tilde{C}}_{R}^{*}}{\beta }\right)C_{R} \end{array}$$

(9d) $$\tilde{I}=n\tilde{F}{\tilde{D}}_{O}\;\left(\frac{k_{f}\;{\tilde{C}}_{O}^{*}-k_{b}{\tilde{C}}_{R}^{*}}{\beta }\right)\;I$$

Here *D̃* is the typical size of the diffusion coefficients of the oxidant and the reductant, *D̃_O_* and *D̃_R_*, and *β* is given by Equation (2). We remark that we have scaled time with the diffusive time-scale associated with the dimensions of the width of the electrode; over time-scales of this order, the diffusion can be regarded as two-dimensional.

Then, in terms of the non-dimensional variables, the problem becomes:

(10a) $$\begin{array}{ccc} D_{O}\nabla^{2}C_{O}=\frac{\partial C_{O}}{\partial t}, & D_{R}\nabla^{2}C_{R}=\frac{\partial C_{R}}{\partial t}, & \;\text{in}\;z>0,t>0 \end{array}$$

(10b) $$\begin{array}{cc} C_{O},C_{R}=0, & \;\text{in}\;z>0,t=0 \end{array}$$

(10c) $$\begin{array}{cc} C_{O},C_{R}\to 0, & \;\text{as}\;x^{2}+z^{2}\to \infty \end{array}$$

(10d) $$\begin{array}{cc} \frac{\partial C_{O}}{\partial z}=\frac{\partial C_{R}}{\partial z}=0, & \;\text{on}|x|>1,z=0 \end{array}$$

(10e) $$\begin{array}{cc} \frac{\partial C_{O}}{\partial z}=\{\begin{array}{l} -\frac{D_{R}}{D_{O}}\frac{\partial C_{R}}{\partial z},\\ k_{f}\;C_{O}-k_{b}C_{R}-\beta , \end{array} & \;\text{on}|x|\leq 1,z=0 \end{array}$$

where *β* is given by Equation (2). The dimensionless Faradaic current per unit axial length through the electrode is given by

(11) $$I(t)=\int_{-1}^{1}\frac{\partial C_{O}}{\partial z}(x,0,t)dx$$

### 2.1. Asymptotic Solution for the Long-time Transient Behaviour

To solve this problem, we take the Laplace transform in time of the expressions detailed in Equation (10) to obtain:

(12a) $$\begin{array}{ccc} D_{O}\nabla^{2}{\bar{C}}_{O}=s{\bar{C}}_{O}, & D_{R}\nabla^{2}{\bar{C}}_{R}=s{\bar{C}}_{R}, & \;\text{in}\;z>0 \end{array}$$

(12b) $$\begin{array}{cc} {\bar{C}}_{O},{\bar{C}}_{R}\to 0, & \;\text{as}\;x^{2}+z^{2}\to \infty \end{array}$$

(12c) $$\begin{array}{cc} \frac{\partial {\bar{C}}_{O}}{\partial z}=\frac{\partial {\bar{C}}_{R}}{\partial z}=0, & \;\text{on}|x|>1,z=0 \end{array}$$

(12d) $$\begin{array}{cc} \frac{\partial {\bar{C}}_{O}}{\partial z}=\{\begin{array}{l} -\frac{D_{R}}{D_{O}}\frac{\partial {\bar{C}}_{R}}{\partial z},\\ k_{f}\;{\bar{C}}_{O}-k_{b}{\bar{C}}_{R}-\frac{\beta }{s}, \end{array} & \;\text{on}|x|\leq 1,z=0 \end{array}$$

The long-time behaviour, *t* ≫ 1, can be deduced from the small *s* behaviour, |*s*| ≪ 1, in Laplace transform space; in dimensional terms, this corresponds to time-scales such that *t̃* ≫ *d̃*^2^/*D̃*. Since the term *β*/*s* in the redox reaction boundary condition on the electrode, detailed in Equation (12d), blows up as *s* → 0, we re-scale the problem by setting *C̅_O,R_* = *c̄_O,R_*/*s*, which gives:

(13a) $$\begin{array}{ccc} D_{O}\nabla^{2}{\bar{c}}_{O}=s{\bar{c}}_{O}, & D_{R}\nabla^{2}{\bar{c}}_{R}=s{\bar{c}}_{R}, & \;\text{in}\;z>0 \end{array}$$

(13b) $$\begin{array}{cc} {\bar{c}}_{O},{\bar{c}}_{R}\to 0, & \;\text{as}\;x^{2}+z^{2}\to \infty \end{array}$$

(13c) $$\begin{array}{cc} \frac{\partial {\bar{c}}_{O}}{\partial z}=\frac{\partial {\bar{c}}_{R}}{\partial z}=0, & \;\text{on}|x|>1,z=0 \end{array}$$

(13d) $$\begin{array}{cc} \frac{\partial {\bar{c}}_{O}}{\partial z}=\{\begin{array}{l} -\frac{D_{R}}{D_{O}}\frac{\partial {\bar{c}}_{R}}{\partial z},\\ k_{f}\;{\bar{c}}_{O}-k_{b}{\bar{c}}_{R}-\beta , \end{array} & \;\text{on}|x|\leq 1,z>0 \end{array}$$

We use the method of matched asymptotic expansions to solve the problem given by Equation (13) approximately for |*s*| ≪ 1. In an inner region near the electrode, over *O*(1) length-scales, the *O*(*s*) terms on the right-hand side of the governing equations in Equation (13a) can be neglected. However these terms cannot be neglected in an outer region when the length-scales are *O*(*s^−^*^1/2^), such that

(14) $$\begin{array}{cc} x=X/\sqrt{s}, & z=Z/\sqrt{s} \end{array}$$

In this outer region, the terms on both sides of the governing equations in Equation (13a) are of similar size. The method of matched asymptotic expansions requires us to search for solutions in the inner and outer regions, and then to match them in an intermediate region where the two solutions have an overlapping region of validity.

In the inner region, we denote the approximate solutions by *h̅_O,R_*(*x*, *z*, *s*), and they satisfy Laplace's equation and the boundary conditions on the surface of the electrode and the insulator:

(15a) $$\begin{array}{ccc} \nabla^{2}{\bar{h}}_{O}=0, & \nabla^{2}{\bar{h}}_{R}=0, & \;\text{in}\;z>0 \end{array}$$

(15b) $$\begin{array}{cc} \frac{\partial {\bar{h}}_{O}}{\partial z}=\frac{\partial {\bar{h}}_{R}}{\partial z}=0, & \;\text{on}|x|>1,z=0 \end{array}$$

(15c) $$\begin{array}{cc} \frac{\partial {\bar{h}}_{O}}{\partial z}=\{\begin{array}{l} -\frac{D_{R}}{D_{O}}\frac{\partial {\bar{h}}_{R}}{\partial z},\\ k_{f}\;{\bar{h}}_{O}-k_{b}{\bar{h}}_{R}-\beta , \end{array} & \;\text{on}|x|\leq 1,z>0 \end{array}$$

In the outer region, we denote the approximate solutions by *H̅_O,R_*(*X*, *Z*, *s*), and they satisfy the modified Helmholtz equations:

(16a) $$\begin{array}{ccc} D_{O}\nabla^{2}{\bar{H}}_{O}={\bar{H}}_{O}, & D_{R}\nabla^{2}{\bar{H}}_{R}={\bar{H}}_{R}, & \;\text{in}\;Z>0 \end{array}$$

with boundary conditions

(16b) $$\begin{array}{cc} {\bar{H}}_{O},{\bar{H}}_{R}\to 0, & \;\text{as}\;X^{2}+Z^{2}\to \infty \end{array}$$

Then the problem is solved approximately by matching the inner solutions, *h̅_O,R_*, to the outer solutions *H̅_O,R_*.

The solutions to the inner problem given by Equation (15) have the following form:

(17a) $${\bar{h}}_{O}(x,z,s)\;=\;A_{0}+\sum_{i=1}^{\infty }\frac{O_{i}(x,z)}{\log{\left(\sqrt{s}\right)}^{i}}$$

(17b) $${\bar{h}}_{R}(x,z,s)\;=\;\frac{D_{O}}{D_{R}}\left[B_{0}+\sum_{i=1}^{\infty }\frac{R_{i}(x,z)}{\log{\left(\sqrt{s}\right)}^{i}}\right]$$

where *A*_0_ and *B*_0_ are constants, and the solutions for *O_i_* and *R_i_*, that allow the conservation of matter boundary condition in Equation (15c) to be satisfied, can be written using a Green's function as:

(18) $$O_{i}(x,z)\;=\;\frac{1}{2\pi }\int_{-1}^{1}\frac{\partial O_{i}}{\partial z}(u,0)\log\left({\left(x-u\right)}^{2}+z^{2}\right)du+A_{i}$$

(19) $$R_{i}(x,z)\;=\;-\frac{1}{2\pi }\int_{-1}^{1}\frac{\partial O_{i}}{\partial z}(u,0)\log\left({\left(x-u\right)}^{2}+z^{2}\right)\text{d}u+B_{i}$$

where again *A_i_* and *B_i_* are constants. The redox reaction boundary condition on the electrode surface given by Equation (15c) implies that:

(20) $$k_{f}\;A_{0}-k_{b}\frac{D_{O}}{D_{R}}B_{0}=\beta$$

and that the following integral equations must be satisfied on the surface of the electrode for *i* ≥ 1:

(21) $$\begin{array}{cc} \frac{\partial O_{i}}{\partial z}(x,0)=\frac{\beta }{\pi }\int_{-1}^{1}\frac{\partial O_{i}}{\partial z}(u,0)\log|x-u|\text{d}u+k_{f}\;A_{i}-k_{b}\frac{D_{O}}{D_{R}}B_{i}, & \;\text{for}|x|\leq 1 \end{array}$$

The solutions to the outer problem in Equation (16) are given by (writing *R*^2^ = *X*^2^ + *Z*^2^):

(22a) $${\bar{H}}_{O}(X,Z,s)\;=\;\sum_{i=1}^{\infty }\frac{C_{i}}{\log{\left(\sqrt{s}\right)}^{i}}K_{0}\;\left(\frac{R}{\sqrt{D_{O}}}\right)$$

(22b) $${\bar{H}}_{R}(X,Z,s)\;=\;\frac{D_{O}}{D_{R}}\sum_{i=1}^{\infty }\frac{D_{i}}{\log{\left(\sqrt{s}\right)}^{i}}K_{0}\;\left(\frac{R}{\sqrt{D_{R}}}\right)$$

where *C_i_* and *D_i_* are constants, and *K*_0_(·) is a modified Bessel function of the second kind [30]. We remark that the branch cut of $\sqrt{s}$ in the relation expressed in Equation (14) must be taken along the negative real axis, so that the vanishing boundary conditions given by Equation (16b) at infinity are satisfied. The unknown constants in the inner and outer solutions, *A_i_*, *B_i_*, *C_i_* and *D_i_*, must be found by asymptotic matching.

We match the inner and outer solutions using the intermediate variable technique [31]:

(23) $$\eta =r{\sqrt{s}}^{\alpha }=R{\sqrt{s}}^{-(1-\alpha )}$$

where *r*^2^ = *x*^2^ + *z*^2^ and 0 < *α* < 1. Written in terms of the intermediate variable *η*, the functions *O_i_* and *R_i_* in the inner solution detailed in Equation (17), and given by the expressions in Equations (18) and (19), become:

(24) $$O_{i}=\frac{1}{\pi }(\log\eta -\alpha \log\sqrt{s})\int_{-1}^{1}\frac{\partial O_{i}}{\partial z}(u,0)\text{d}u+A_{i}+O({\sqrt{s}}^{\alpha })$$

(25) $$R_{i}=-\frac{1}{\pi }(\log\eta -\alpha \log\sqrt{s})\int_{-1}^{1}\frac{\partial O_{i}}{\partial z}(u,0)\text{d}u+B_{i}+O({\sqrt{s}}^{\alpha })$$

whilst the modified Bessel functions in the outer solutions expressed in Equation (22) become:

(26) $$K_{0}\left(\frac{R}{\sqrt{D_{O}}}\right)=-\gamma -(1-\alpha )\log\sqrt{s}-\log\eta +\log\sqrt{D_{O}}+\log2+O(s^{\left(1-\alpha \right)}\log s)$$

(27) $$K_{0}\left(\frac{R}{\sqrt{D_{R}}}\right)=-\gamma -(1-\alpha )\log\sqrt{s}-\log\eta +\log\sqrt{D_{R}}+\log2+O(s^{\left(1-\alpha \right)}\log s)$$

where *γ* is Euler's constant.

In the intermediate region, therefore, the inner expansion can be written:

(28a) $${\bar{h}}_{O}\sim A_{0}-\frac{\alpha }{\pi }\int_{-1}^{1}\frac{\partial O_{1}}{\partial z}(u,0)\text{d}u+\sum_{i=1}^{\infty }\;\left(-\frac{\alpha }{\pi }\int_{-1}^{1}\frac{\partial O_{i+1}}{\partial z}(u,0)\text{d}u+\frac{\log\eta }{\pi }\int_{-1}^{1}\frac{\partial O_{i}}{\partial z}(u,0)\text{d}u+A_{i}\right){\left(\log\sqrt{s}\right)}^{-i}$$

(28b) $${\bar{h}}_{R}\sim \frac{D_{O}B_{O}}{D_{R}}+\frac{\alpha D_{O}}{\pi D_{R}}\int_{-1}^{1}\frac{\partial O_{1}}{\partial z}(u,0)\text{d}u-\frac{D_{O}}{D_{R}}\sum_{i=1}^{\infty }\;\left(-\frac{\alpha }{\pi }\int_{-1}^{1}\frac{\partial O_{i+1}}{\partial z}(u,0)\text{d}u+\frac{\log\eta }{\pi }\int_{-1}^{1}\frac{\partial O_{i}}{\partial z}(u,0)\text{d}u-B_{i}\right)\;{\left(\log\sqrt{s}\right)}^{-i}$$

and the outer expansion becomes:

(29a) $${\bar{H}}_{O}\sim -C_{i}(1-\alpha )+\sum_{i=1}^{\infty }\left(C_{i}(\log2-\gamma -\log\eta +\log\sqrt{D_{O}})-(1-\alpha )C_{i+1}\right)\;{\left(\log\sqrt{s}\right)}^{-i}$$

(29b) $${\bar{H}}_{R}\sim \frac{D_{1}D_{O}}{D_{R}}(1-\alpha )+\frac{D_{O}}{D_{R}}\sum_{i=1}^{\infty }\left(D_{i}(\log2-\gamma -\log\eta +\log\sqrt{D_{R}})-(1-\alpha )D_{i+1}\right)\;{\left(\log\sqrt{s}\right)}^{-i}$$

Comparing similar powers of ${\left(\log\sqrt{s}\right)}^{-i}$ in Equations (28) and (29) gives at *O*(1):

(30) $$A_{0}-\frac{\alpha }{\pi }\int_{-1}^{1}\frac{\partial O_{1}}{\partial z}(u,0)\text{d}u\;=\;-C_{1}(1-\alpha )$$

(31) $$B_{0}+\frac{\alpha }{\pi }\int_{-1}^{1}\frac{\partial O_{1}}{\partial z}(u,0)\text{d}u\;=\;-D_{1}(1-\alpha )$$

which implies that:

(32) $$A_{0}=-B_{0}=-C_{1}=D_{1}=\frac{1}{\pi }\int_{-1}^{1}\frac{\partial O_{1}}{\partial z}(u,0)\text{d}u$$

Using the condition given by Equation (20), we find that:

(33) $$\frac{1}{\pi }\int_{-1}^{1}\frac{\partial O_{1}}{\partial z}(u,0)\text{d}u=1$$

so that

(34) $$A_{0}=-B_{0}=-C_{1}=D_{1}=1$$

At $O{\left(\log\sqrt{s}\right)}^{-i}$ (*i* = 1, 2, 3, …), the matching process between Equations (28) and (29) gives:

(35) $$-\frac{\alpha }{\pi }\int_{-1}^{1}\frac{\partial O_{i+1}}{\partial z}(u,0)\text{d}u+\frac{\log\eta }{\pi }\int_{-1}^{1}\frac{\partial O_{i}}{\partial z}(u,0)\text{d}u+A_{i}=C_{i}(\log2-\gamma -\log\eta +\log\sqrt{D_{O}})-(1-\alpha )C_{i+1}$$

(36) $$\frac{\alpha }{\pi }\int_{-1}^{1}\frac{\partial O_{i+1}}{\partial z}(u,0)\text{d}u-\frac{\log\eta }{\pi }\int_{-1}^{1}\frac{\partial O_{i}}{\partial z}(u,0)\text{d}u+B_{i}=D_{i}(\log2-\gamma -\log\eta +\log\sqrt{D_{R}})-(1-\alpha )D_{i+1}$$

Equations (35) and (36) imply firstly that:

(37) $$\begin{array}{cc} C_{i}=-D_{i}=-\frac{1}{\pi }\int_{-1}^{1}\frac{\partial O_{i}}{\partial z}(u,0)\text{d}u, & \;\text{for}\;i\geq 1 \end{array}$$

and, secondly, that:

(38) $$\begin{array}{cc} \begin{array}{c} C_{i+1}\;=\;C_{i}(\log2-\gamma +\log\sqrt{D_{O}})-A_{i},\\ D_{i+1}\;=\;D_{i}(\log2-\gamma +\log\sqrt{D_{R}})-B_{i}, \end{array}\} & \text{for}\;i\geq 1 \end{array}$$

Now we note that the conditions given by Equations (32) and (37) provide solvability conditions for the integral equations in Equation (21), namely that:

(39) $$\begin{array}{cc} \frac{1}{\pi }\int_{-1}^{1}\frac{\partial O_{i}}{\partial z}(u,0)\text{d}u=-C_{i}, & \;\text{for}\;i\geq 1 \end{array}$$

Writing

(40) $$\frac{\partial O_{i}}{\partial z}(x,0)=\beta^{-1}\left(k_{f}\;A_{i}-k_{b}\frac{D_{O}}{D_{R}}B_{i}\right)\;F(x)$$

then the integral equations in Equation (21) and solvability conditions given by Equation (39) become:

(41) $$\begin{array}{cc} F(x)=\frac{\beta }{\pi }\int_{-1}^{1}F(u)\log|x-u|\text{d}u+\beta , & \;\text{for}|x|\leq 1 \end{array}$$

with

(42) $$\frac{1}{\pi }\int_{-1}^{1}F(u)\text{d}u=\frac{1}{Q(\beta )}$$

where

(43) $$Q(\beta )=-\beta^{-1}C_{i}^{-1}\left(k_{f}\;A_{i}-k_{b}\frac{D_{O}}{D_{R}}B_{i}\right)$$

Solution of the integral equation given in Equation (41) for *F*(*x*) and substitution into Equation (42) determines *Q*(*β*) as a function of *β*, and then Equation (43) gives another relationship between *A_i_*, *B_i_* and *C_i_*.

Eliminating *A_i_*, *B_i_* and *D_i_* from Equations (37), (38) and (43), we find a recurrence relation for *C_i_*:

(44) $$C_{i+1}=\left(\log2-\gamma +Q(\beta )+\log\sqrt{D_{O}}+k_{b}\beta^{-1}\frac{D_{O}}{D_{R}}\log\sqrt{\frac{D_{O}}{D_{R}}}\right)C_{i}$$

with initial condition *C*_1_ = −1 from Equation (34). This has solution for *i* ≥ 1 given by:

(45) $$C_{i}=-{\left(\log2-\gamma +Q(\beta )+\log\sqrt{D_{O}}+k_{b}\beta^{-1}\frac{D_{O}}{D_{R}}\log\sqrt{\frac{D_{O}}{D_{R}}}\right)}^{i-1}$$

Finally, the Laplace transform of the current per unit axial length, *Ī*(*s*), *is* given by the following:

(46) $$\bar{I}(s)\sim \frac{1}{s}\int_{-1}^{1}\frac{\partial {\bar{h}}_{O}}{\partial z}(u,0,s)\text{d}u$$

(47) $$\sim \frac{1}{s}\sum_{i=1}^{\infty }\int_{-1}^{1}\frac{\partial O_{i}}{\partial z}(u,0)\text{d}u{\left(\log\sqrt{s}\right)}^{-i}$$

(48) $$\sim -\frac{\pi }{s}\sum_{i=1}^{\infty }C_{i}{\left(\log\sqrt{s}\right)}^{-i}$$

where we have used the expression for *h̄_O_* given in Equation (17a), and have employed the relationship given in Equation (39). Substitution of the solution for *C_i_* detailed in Equation (45) into Equation (48) gives:

(49) $$\bar{I}(s)\sim \frac{\pi }{s}\sum_{i=1}^{\infty }{\left(\log2-\gamma +Q(\beta )+\log\sqrt{D_{O}}+k_{b}\beta^{-1}\frac{D_{O}}{D_{R}}\log\sqrt{\frac{D_{R}}{D_{O}}}\right)}^{i-1}{\left(\log\sqrt{s}\right)}^{-i}$$

(50) $$\sim \left(\frac{\pi }{s}\right)\frac{1}{\log\sqrt{s}-\left(\log2-\gamma +Q(\beta )+\log\sqrt{D_{O}}+k_{b}\beta^{-1}\frac{D_{O}}{D_{R}}\log\sqrt{\frac{D_{R}}{D_{O}}}\right)}$$

This can be re-written in similar form to the solution in Phillips [26]:

(51) $$\bar{I}(s)\sim -\left(\frac{2\pi }{s}\right)\frac{1}{\log(s^{-1})+\phi +\log D_{O}}$$

where *ϕ* is given by:

(52) $$\phi =2(\log2-\gamma +Q(\beta ))+k_{b}\beta^{-1}\frac{D_{O}}{D_{R}}\log\left(\frac{D_{R}}{D_{O}}\right)$$

Then Phillips [26] shows that this can be inverted to give the long-time current as:

(53) $$I(t)\sim -2\pi ℐ(e^{\phi }D_{O}t)$$

where

(54) $$ℐ(a)=\int_{0}^{\infty }\frac{\exp(-au)}{u\left({\left(\log u\right)}^{2}+\pi^{2}\right)}\text{d}u$$

Integration by parts (to remove the integrable singularity at *u* = 0), and the change of variable *υ* = *au*, casts Equation (54) into a more amenable form for numerical evaluation:

(55) $$ℐ(a)=\frac{1}{2}+\frac{1}{\pi }\int_{0}^{\infty }e^{-\upsilon }\arctan\left[\frac{1}{\pi }\log\left(\upsilon /a\right)\right]\text{d}\upsilon$$

Phillips [26] tabulated values of the function ℐ(*a*) and showed that it can be expanded asymptotically in inverse powers of the logarithm for large *a*, such that

(56) $$ℐ(a)={\left(\log a\right)}^{-1}-\gamma {\left(\log a\right)}^{-2}-\left(\frac{\pi^{2}}{6}-\gamma^{2}\right){\left(\log a\right)}^{-3}+O{\left(\log a\right)}^{-4}$$

where *γ* is Euler's constant. Although this expression is simpler to implement, Phillips [26] emphasises that the expression in Equation (54), or equivalently Equation (55), is more accurate as it incorporates all the logarithmic terms, so that the error in the current is algebraic rather than logarithmic.

In dimensional terms, the expression in Equation (53) for the Faradaic current per unit axial length (A·m^−1^) through the microband electrode becomes:

(57a) $$\tilde{I}(\tilde{t})\sim -2\pi n\tilde{F}{\tilde{D}}_{O}\left(\frac{{\tilde{C}}_{O}^{*}-\frac{{\tilde{k}}_{b}}{{\tilde{k}}_{f}}{\tilde{C}}_{R}^{*}}{1+\frac{{\tilde{k}}_{b}{\tilde{D}}_{O}}{{\tilde{k}}_{f}\;{\tilde{D}}_{R}}}\right)\;ℐ\;\left(e^{\phi }{\tilde{D}}_{O}\tilde{t}/{\tilde{d}}^{2}\right)$$

where ℐ(·) is evaluated from Equation (55) and *ϕ* is given by:

(57b) $$\phi =2(\log2-\gamma +Q(\beta ))+{\left(1+\frac{{\tilde{D}}_{R}{\tilde{k}}_{f}}{{\tilde{D}}_{O}{\tilde{k}}_{b}}\right)}^{-1}\log\;\left(\frac{{\tilde{D}}_{R}}{{\tilde{D}}_{O}}\right)$$

We recall that *β* is given by Equation (2), and the function *Q*(*β*) in Equation (57b) must be calculated numerically by solving the integral equation in Equation (41) for *F*(*x*) and substituting the result into the expression in Equation (42). In order that the experimentalist does not have to perform these numerical calculations themselves, we have provided a working curve for *Q*(*β*) over the range 10^−2^ ≤ *β* ≤ 10^3^ as the file “ Q_beta_working_curve.csv” in the Supplementary Information. The calculation of this working curve is detailed in the next section, where we also provide asymptotic analytical expressions for small and large *β*, given by Equations (62) and (63), which can be used to calculate *Q*(*β*) outside the range of the working curve.

We remark that the Faradaic current per unit axial length through a laminar electrode in free space is simply double the expression in Equation (57a), since the current is identical through both sides of the lamina.

### 2.2. Calculation of Q(β)

It is useful for the experimentalist to have a working curve to evaluate the function *Q*(*β*) in Equation (57), and in this section we describe how we have calculated it. We also provide analytical asymptotic expressions that can be used to calculate *Q*(*β*) for small and large *β*.

For a particular value of *β*, *Q*(*β*) is calculated by solving the integral equation in Equation (41) for *F*(*x*) and inserting the result into the expression in Equation (42). We solved the integral equation by removing the logarithmic singularity and applying the Nystrom method (*cf.* Delves and Mohamed [32]). Consider *N* Gauss–Legendre quadrature weights, *w_i_*, at the abscissae *x_i_* in the interval (−1, 1). At each point *x_i_*, the logarithmic singularity in the integral equation detailed in Equation (41) can be removed by writing it as:

(58) $$\begin{array}{ll} F(x_{i})= & \frac{\beta }{\pi }\int_{-1}^{1}(F(s)-F(x_{i}))\log|x_{i}-s|\text{d}s+\frac{\beta }{\pi }\left((1-x_{i})\log(1-x_{i})+(1+x_{i})\log(1+x_{i})-2\right)F(x_{i})+\beta ,\;\text{for}\;i=1,\ldots ,N \end{array}$$

The integrals in Equation (58) can be evaluated using Gauss-Legendre quadrature at the same abscissae to give:

(59) $$F(x_{i})=\frac{\beta }{\pi }\sum_{k=1}^{i-1}w_{k}\left(F(x_{k})-F(x_{i})\right)\log|x_{i}-x_{k}|+\frac{\beta }{\pi }\sum_{k=i+1}^{N}w_{k}\left(F(x_{k})-F(x_{i})\right)\log|x_{i}-x_{k}|+\frac{\beta }{\pi }\left((1-x_{i})\log(1-x_{i})+(1+x_{i})\log(1-x_{i})-2\right)F(x_{i})+\beta$$

with appropriate care taken whenever *i* = 1 or *i* = *N*. This represents *N* linear equations to be solved for the *N* unknowns *F*(*x_i_*). Then *Q*(*β*) can be calculated from Equation (42) as follows:

(60) $$Q(\beta )=\frac{\pi }{\sum_{i=1}^{N}w_{i}F(x_{i})}$$

For each *β*, we calculated *Q*(*β*) using *N* = 400 Gauss–Legendre weights. The values of *β* used to calculate the points on the working curve for *Q*(*β*) were logarithmically spaced between 0.01 and 1000 as follows:

(61) $$\begin{array}{cc} \beta_{j}=10^{-2+5j/2000}, & j=0,\ldots ,2000 \end{array}$$

Convergence was slower for larger *β_j_*, and the maximum absolute change in *Q*(*β_j_*) on doubling *N* from 200 to 400 was less than 10^−6^ at *β_j_* = 1000. The working curve is attached in the Supplementary Information as a CSV file named “ Q_beta_working_curve.csv”.

Asymptotic expressions can also be derived for small and large *β*. For small *β*, the function *Q*(*β*) takes the following form:

(62) $$Q(\beta )=\frac{\pi }{2\beta }+\frac{3}{2}-\log2+O(\beta )$$

For large *β*, the asymptotic solution is given by (proof in the Appendix):

(63) $$Q(\beta )=\log2+\frac{1}{\pi \beta }\log\beta +o(\beta^{-1}\log\beta )$$

**Figure 3.**
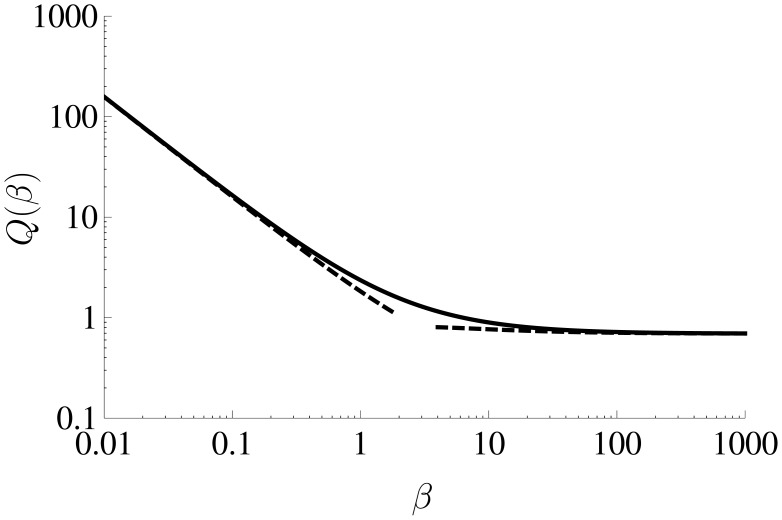
Log-log plot of the function *Q*(*β*). The solid line has been calculated numerically as described in Section 2.2, and the dashed lines have been plotted using the asymptotic expressions given by Equation (62) for *β* ≪ 1, and Equation (63) for *β* ≫ 1.

We include a log-log plot of *Q*(*β*) in Figure 3, which shows that the agreement between the asymptotic solutions (dashed lines) and the numerically calculated values (solid lines) is good. For *β* ≲ 0.01, the expression in Equation (62) can be used to calculate *Q*(*β*), while for *β* ≳ 1000, the expression in Equation (63) provides a reasonable approximation. For all other values, the numerically calculated working curve can be used.

## 3. Results and Discussion

In the previous section, we have derived the asymptotic solution detailed in Equation (57) for the long-time-dependent chronoamperometric current per unit axial length due to two-dimensional diffusion at an inlaid microband electrode. The solution allows for finite kinetics and unequal diffusion coefficients of the oxidant and the reductant. In this section, we show that the expression in Equation (57) reduces to the first term in the series derived by Aoki *et al.* [24] (see also Phillips [26]) for the current in the diffusion-limited regime due to extreme polarization. We also present the simplified form of Equation (57) for reversible reactions; in the case of identical diffusion coefficients, this expression also reduces to the first term in the series derived by Aoki *et al.* [24]. We compare the analytical solution to the results of numerical calculations, and we illustrate that unequal diffusion coefficients can cause significant differences in the current response. Finally, we discuss the formula for the current response due to a one-step, one-electron redox reaction whenever the rate constants are modelled by the Butler–Volmer expressions [1], and we indicate that the width of the electrode must be chosen carefully to allow accurate estimates of the standard kinetic rate constant and the electron transfer coefficient to be obtained from the long-time current response.

### 3.1. Diffusion-limited Currents due to Extreme Polarization

The diffusion-limited reduction current per unit axial length due to extreme polarization corresponds to *k_f_* → ∞ and *k_b_* → 0. This means that *β* → ∞, and the expression in Equation (57) for the current response becomes:

(64) $$\tilde{I}(\tilde{t})\sim -2\pi n\tilde{F}{\tilde{D}}_{O}{\tilde{C}}_{O}^{*}\;ℐ\;\left(16\;e^{-2\gamma }{\tilde{D}}_{O}\tilde{t}/{\tilde{d}}^{2}\right)$$

where we have used Equation (63) to set *Q*(∞) = log 2. This expression agrees with first term in the series derived by Aoki *et al.* [24] (see also Phillips [26]).

Similarly, the diffusion-limited oxidation current per unit axial length due to extreme polarization corresponds to *k_f_* → 0 and *k_b_* → ∞, which gives:

(65) $$\tilde{I}(\tilde{t})\sim -2\pi n\tilde{F}{\tilde{D}}_{R}{\tilde{C}}_{R}^{*}\;ℐ\;\left(16\;e^{-2\gamma }{\tilde{D}}_{R}\tilde{t}/{\tilde{d}}^{2}\right)$$

As expected, this is equivalent to Equation (64) with the sign changed, and the subscripts *O* and *R* interchanged.

### 3.2. Reversible Reactions

For reversible reactions, *k_f_*, *k_b_* → ∞ such that *k_f_*/*k_b_* = *O*(1). In this case, since *β* → ∞ and *Q*(∞) = log 2, we obtain:

(66a) $$\tilde{I}(\tilde{t})\sim -2\pi n\tilde{F}{\tilde{D}}_{O}\left(\frac{{\tilde{C}}_{O}^{*}-\frac{{\tilde{k}}_{b}}{{\tilde{k}}_{f}}{\tilde{C}}_{R}^{*}}{1+\frac{{\tilde{k}}_{b}}{{\tilde{k}}_{f}}\frac{{\tilde{D}}_{O}}{{\tilde{D}}_{R}}}\right)\;ℐ\;\left(e^{\phi }{\tilde{D}}_{O}\tilde{t}/{\tilde{d}}^{2}\right)$$

where

(66b) $$\phi =2\left(2\log2-\gamma \right)+{\left(1+\frac{{\tilde{D}}_{R}{\tilde{k}}_{f}}{{\tilde{D}}_{O}{\tilde{k}}_{b}}\right)}^{-1}\log\left(\frac{{\tilde{D}}_{R}}{{\tilde{D}}_{O}}\right)$$

In the case of identical diffusion coefficients, this expression also reduces to the first term in the series derived by Aoki *et al.* [24], since the logarithmic term in Equation (66b) vanishes.

### 3.3. Comparison with Numerical Calculations

To calculate the current numerically, we employed the fully implicit finite-difference method (FIFD) devised by Gavaghan [33,34] for the inlaid disk electrode, but adapted for the Cartesian coordinate system appropriate to the microband electrode. We simulated the non-dimensional problem detailed in Equation (10) on the finite domain 0 ≤ *x* ≤ *x*_max_ = 201, 0 ≤ *z* ≤ *z*_max_ = 200 for 0 < *t* ≤ *t*_max_ = 100. Considerations of symmetry allowed us to simulate only half the domain (*x* > 0) with extra symmetry boundary conditions ∂*C_O_*/∂*x* = ∂*C_R_*/∂*x* = 0 on *x* = 0. The finite boundaries of the domain, *x*_max_ = 201 and *z*_max_ = 200, were chosen to satisfy the criterion that *x*_max_, $z_{\max}>\max\{6\sqrt{D_{O}t_{\max}},6\sqrt{D_{R}t_{\max}}\}$ (*cf.* [17]); this ensures that application of the conditions *C_O_* = *C_R_* = 0 at the finite boundaries *x*_max_ and *z*_max_, instead of infinity, does not distort the current through the electrode. Gavaghan's method uses a spatial grid that expands exponentially away from the edge of the electrode in order to resolve accurately the large flux in the neighbourhood of the edge. Here we used the same grid parameters as suggested in [33,34]: the spatial steps in the *x*- and *z*-directions nearest the edge of the electrode at *x* = 1 are given by *h*_last_ = 8 × 10^−5^, and the expansion factor is given by *f* = 1.175. The initial time-step was taken to be 10^−5^, and was increased by a factor of 10 every thousand steps.

**Figure 4.**
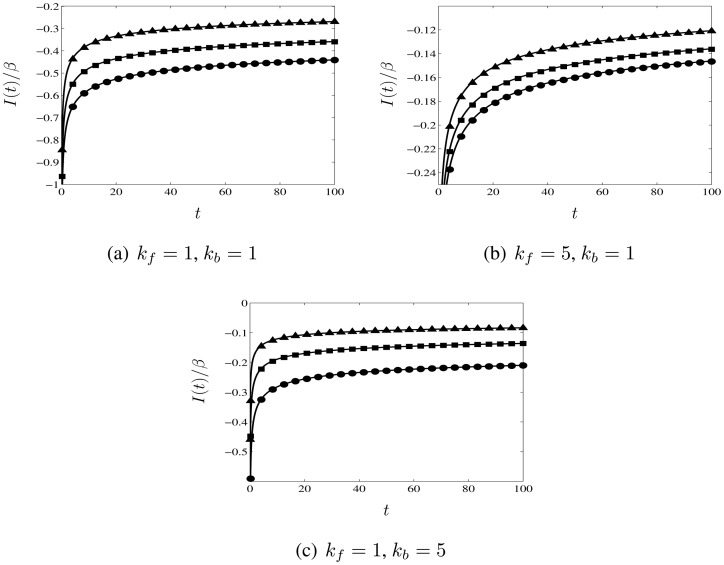
Plots of *I*(*t*)/*β* versus *t* for different rate constants: (**a**) *k_f_* = 1, *k_b_* = 1; (**b**) *k_f_* = 5, *k_b_* = 1; and (**c**) *k_f_* = 1, *k_b_* = 5. The solid lines are calculated using the approximate analytical expression in Equation (53) for the long-time current response, while the symbols are plotted from the results of the numerical calculations described in Section 3.3. The symbols correspond to different ratios of the diffusion coefficients: *D_O_* = 1, *D_R_* = 0.5 (triangles), *D_O_* = 1, *D_R_* = 1 (squares), and, *D_O_* = 1, *D_R_* = 2 (circles). It is apparent from the plots that the effect of unequal diffusion coefficients can be significant.

We plot *I*(*t*)/*β* in Figure 4(a-c) for 0 < *t* ≤ 100 for different rate constants: (a) *k_f_* = 1, *k_b_* = 1, (b) *k_f_* = 5, *k_b_* = 1, and (c) *k_f_* = 1, *k_b_* = 5. We have divided the current by *β* to capture completely the effect of varying the ratio of the diffusion coefficients (*cf.* the factor of *β*^−1^ in the non-dimensionalisation of the current in Equation (9d)). We note that *β* (given by Equation (2)) can be written in terms of the non-dimensional variables as:

(67) $$\beta =k_{f}+k_{b}\frac{D_{O}}{D_{R}}.$$

**Figure 5.**
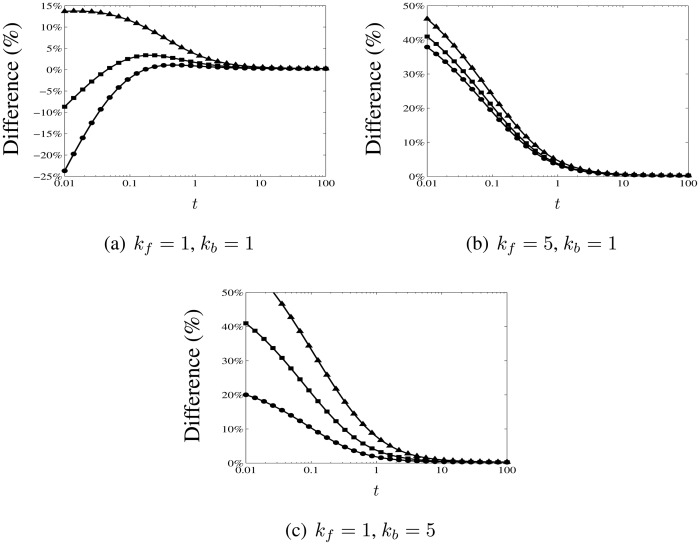
Log-linear plots of the time-varying percentage difference between the analytical expression given by Equation (53) for the current response and numerical results calculated as described in Section 3.3, using the same parameters as the plots in Figure 4: (**a**) *k_f_* = 1, *k_b_* = 1, (**b**) *k_f_* = 5, *k_b_* = 1, and (**c**) *k_f_* = 1, *k_b_* = 5. The symbols correspond to different ratios of the diffusion coefficients: *D_O_* = 1, *D_R_* = 0.5 (triangles), *D_O_* = 1, *D_R_* = 1 (squares), and, *D_O_* = 1, *D_R_* = 2 (circles). The analytical expression approaches the numerical results for longer times, when it becomes valid. For *t* ≥ 5, the maximum difference is less than 1.75% for all the parameters considered.

The different symbols in each plot correspond to different ratios of the diffusion coefficients: *D_O_* = 1, *D_R_* = 0.5 (triangles); *D_O_* = 1, *D_R_* = 1 (squares); and, *D_O_* = 1, *D_R_* = 2 (circles). The solid lines are calculated using the analytical expression in Equation (53) for the non-dimensional long-time current response, with *ϕ* given by Equation (52) and the integral ℐ(*a*) evaluated using the expression in Equation (55), while the symbols are plotted from the numerical results. It is apparent from Figure 4(a-c) that unequal diffusion coefficients can substantially alter the magnitude of the current response. In Figure 5 (a-c), we plot the percentage difference between the numerical results and the analytical expression as a function of time for the same parameters. As expected the analytical expression approaches the numerical results more closely for longer times, since this is when it becomes valid. For *t* ≥ 5, the maximum difference is less than 1.75% for all the parameters considered. (We also remark that comparison with the reference values calculated by Britz *et al.* [18] for the diffusion-limited current indicates a similar level of accuracy; for *t* ≥ 5, the percentage difference in this regime is less than 1%.)

### 3.4. Long-time Current Response for a One-step, One-electron Redox Reaction Modelled in the Butler–Volmer Framework

So far we have not assumed any model for the rate constants of the redox reaction, *k̃_f_* and *k̃_b_*. For a one-step, one-electron process, so that *n* = 1 for the redox reaction detailed in Equation (1), the Butler–Volmer model [1] describes the rate constants as an exponential function of the potential *Ẽ* (V) at the electrode surface:

(68) $$\begin{array}{cc} {\tilde{k}}_{f}={\tilde{k}}_{0}e^{-\alpha \frac{\tilde{F}}{\tilde{R}\tilde{T}}(\tilde{E}-{\tilde{E}}_{0})}, & {\tilde{k}}_{b}={\tilde{k}}_{0}e^{\left(1-\alpha )\frac{\tilde{F}}{\tilde{R}\tilde{T}}(\tilde{E}-{\tilde{E}}_{0}\right)} \end{array}$$

where *k̃*_0_ (m·s^−1^) is the standard kinetic rate constant, 0 < *α* < 1 is the electron transfer coefficient, and *Ẽ*_0_ (V) is the formal oxidation potential. The remaining parameters are Faraday's constant, *F̃* (96,485 C·mol^−1^), the universal gas constant, *R̃* (8.3145 J·K^−1^·mol^−1^), and the temperature, *T̃* (K). Substitution of this form of *k̃_f_* and *k̃_b_* into Equation (57), and setting *n* = 1, gives the Butler–Volmer long-time current response as:

(69a) $$\tilde{I}(\tilde{t})\sim -2\pi n\tilde{F}{\tilde{D}}_{O}\left(\frac{{\tilde{C}}_{O}^{*}-e^{\frac{\tilde{F}}{\tilde{R}\tilde{T}}(\tilde{E}-{\tilde{E}}_{0})}{\tilde{C}}_{R}^{*}}{1+e^{\frac{\tilde{F}}{\tilde{R}\tilde{T}}(\tilde{E}-{\tilde{E}}_{0})}\frac{{\tilde{D}}_{O}}{{\tilde{D}}_{R}}}\right)\;ℐ\;\left(e^{\phi }{\tilde{D}}_{O}\tilde{t}/{\tilde{d}}^{2}\right)$$

where

(69b) $$\phi =2(\log2-\gamma +Q(\beta )){\left(1+e^{-\frac{\tilde{F}}{\tilde{R}\tilde{T}}(\tilde{E}-{\tilde{E}}_{0})}\frac{{\tilde{D}}_{R}}{{\tilde{D}}_{O}}\right)}^{-1}\log\left(\frac{{\tilde{D}}_{R}}{{\tilde{D}}_{O}}\right)$$

and *β*, given by Equation (2), becomes:

(69c) $$\beta =\frac{{\tilde{k}}_{0}\tilde{d}}{{\tilde{D}}_{O}}\left\{e^{-\alpha \frac{\tilde{F}}{\tilde{R}\tilde{T}}(\tilde{E}-{\tilde{E}}_{0})}+e^{\left(1-\alpha )\frac{\tilde{F}}{\tilde{R}\tilde{T}}(\tilde{E}-{\tilde{E}}_{0}\right)}\left(\frac{{\tilde{D}}_{O}}{{\tilde{D}}_{R}}\right)\right\}$$

It is interesting to observe from Equation (69) that the only impact of the standard kinetic rate constant, *k̃*_0_, and the electron transfer coefficient, *α*, on the long-time current response occurs through the function *Q*(*β*). The sensitivity of *Q*(*β*) to the parameters *k̃*_0_ and *α* depends on its gradient, which tends to zero as *β* → ∞ (see the asymptotic expression in Equation (63) and Figure 3). In addition, as *β* → 0, the current response, and its sensitivity to *k̃*_0_ and *α*, also tends to zero. This suggests that there is a range of *β* over which the sensitivity to the parameters *k̃*_0_ and *α* is significant enough to allow them to be estimated accurately using the long-time current response; outside this range the sensitivity to the parameters will be too small. Since the size of *β* can be controlled by changing the width of the microband *d̃* (*cf.* Equation (69c)), this implies that the size of the electrode should be chosen such that *β* lies within this sensitive range. However, as this range will depend on all of the system parameters, the appropriate size of *d̃* can only be estimated before the experiment using a priori estimates of each of the parameters. It can then be verified a posteriori that the sensitivity of the current response was significant enough to provide accurate estimates of the parameters, e.g., by calculating confidence limits. We intend to explore the choice of electrode size more carefully in a future article.

## 4. Conclusions

We have derived the approximate asymptotic expression given in Equation (57) for the long-time chronoamperometric current response at an inlaid microband electrode whose axial length is much larger than its width so that the diffusion is effectively two-dimensional. For a laminar electrode in free space, the current response is simply double the expression given in Equation (57). The solution allows for finite redox kinetics and unequal diffusion coefficients of the oxidant and reductant. As an input, it requires calculation of a function *Q*(*β*), which we have determined numerically and provided as a working curve in the Supplementary Information. We have also provided simple asymptotic expressions that can be used to calculate *Q*(*β*) whenever *β* is small or large. The expression for the current response has been validated by comparison with the results of numerical calculations, and we have demonstrated that the effect of unequal diffusion coefficients on the current response can be substantial. Finally, we have discussed the form of the long-time current response due to a one-step, one-electron redox reaction when the rate constants are modelled using the Butler–Volmer framework, so that the current response is given by the expression in Equation (69). The formula indicates the importance of choosing the width of the microband carefully to ensure accurate estimation of the standard kinetic rate constant and the electron transfer coefficient from the long-time current response. We intend to investigate this more thoroughly in a future article.

## Appendix

### A. Large-*β* Asymptotic Solution for *Q*(*β*)

To derive the large-*β* asymptotic solution for *Q*(*β*), we employ a similar argument to that used by Phillips [35] to determine the asymptotic solution for near-diffusion-limited currents at inlaid electrodes when the diffusion is three-dimensional.

As described in the main body of the article, the function *Q*(*β*) can be found by solving the integral equation detailed in Equation (41) and substituting the result into Equation (42). However, to proceed with the large-*β* asymptotics, we consider an alternative, but equivalent, formulation of the problem. The function *Q*(*β*) can be also be determined by finding the constant coefficient as *r* → ∞ (which is equal to *Q*(*β*) in the solution to the following boundary value problem:

(70a)
$$\begin{array}{cc} \nabla^{2}G=0, & \;\text{in}\;z>0 \end{array}$$

(70b)
$$\begin{array}{cc} \frac{\partial G}{\partial z}=0, & \;\text{on}|x|>1,z=0 \end{array}$$

(70c)
$$\begin{array}{cc} \frac{\partial G}{\partial z}=\beta G, & \;\text{on}|x|\leq 1,z=0 \end{array}$$

(70d)
$$\begin{array}{cc} G\sim \log r, & \;\text{as}\;r\to \infty \end{array}$$

As *β* → ∞, the leading-order solution to the problem in Equation (70), denoted by *G*_∞_, satisfies:

(71a)
$$\begin{array}{cc} \nabla^{2}G_{\infty }=0, & \;\text{in}\;z>0 \end{array}$$

(71b)
$$\begin{array}{ll} \frac{\partial G_{\infty }}{\partial z}=0, & \;\text{on}|x|>1,z=0 \end{array}$$

(71c)
$$\begin{array}{ll} G_{\infty }=0, & \;\text{on}|x|\leq 1,z=0 \end{array}$$

(71d)
$$\begin{array}{cc} G_{\infty }\sim \log r, & \;\text{as}\;r\to \infty \end{array}$$

with solution derived using conformal mapping (see [26]) to be:

(72)
G∞=Relog(x+Iz+((x+Iz)2−1)12)

Finding the constant coefficient in this solution as *r* → ∞ implies that:

(73)
Q(∞)=log2

To find higher order corrections to this expression, we employ a similar technique to Phillips [35], and use Green's second identity to write:

(74)
$$\int_{-1}^{1}(1-G)\frac{\partial G_{\infty }}{\partial z}-(1-G_{\infty })\frac{\partial G}{\partial z}\text{d}x=\int_{\Gamma_{R}}(1-G)\frac{\partial G_{\infty }}{\partial n}-(1-G_{\infty })\frac{\partial G}{\partial n}\text{d}l$$

where *G* is the solution to the entire problem given in Equation (70). Here the integral on the left-hand side is along the surface of the electrode on *z* = 0, while, on the right-hand side, the integral is around a semi-circle of radius *R* in *z* > 0 centred on the origin (denoted by Γ*_R_*) and ∂/∂*n* denotes the outward normal derivative. Now, the condition that *G*, *G*_∞_ ∼ log *r* as *r* → ∞ implies that:

(75)
$$\begin{array}{cc} \int_{-1}^{1}\frac{\partial G_{\infty }}{\partial z}\text{d}x=\pi , & \int_{-1}^{1}\frac{\partial G}{\partial z}\text{d}x=\pi \end{array}$$

Also, on the surface of the electrode:

(76)
$$\begin{array}{ccc} G_{\infty }=0, & G=\frac{1}{\beta }\frac{\partial G}{\partial z}, & \text{on}|x|\leq 1,z=0 \end{array}$$

and, in the far-field:

(77)
$$\begin{array}{ccc} G_{\infty }\sim \log r+\log2, & G\sim \log r+Q(\beta ), & \text{as}\;r\to \infty \end{array}$$

Substituting the expressions in Equations (75)–(77) into Equation (74), and letting *R* → ∞, we find that:

(78)
$$Q(\beta )=\log2+\frac{1}{\pi \beta }\int_{-1}^{1}\frac{\partial G}{\partial z}\frac{\partial G_{\infty }}{\partial z}\text{d}x$$

The leading-order solution for *G* is *G*_∞_, and this breaks down as a singular perturbation in an *O*(*β*^−1^) region of *x* = ±1. Taking 1 ≫ *δ* ≫ *β*^−1^, and using symmetry about *x* = 0, we can write the integral on the right-hand side of Equation (78) as:

(79)
$$\int_{-1}^{1}\frac{\partial G}{\partial z}\frac{\partial G_{\infty }}{\partial z}\text{d}x\sim 2\int_{0}^{1-\delta }{\left(\frac{\partial G_{\infty }}{\partial z}\right)}^{2}\text{d}x+2\int_{1-\delta }^{1}\frac{\partial G}{\partial z}\frac{\partial G_{\infty }}{\partial z}\text{d}x$$

Since

(80)
$$\begin{array}{cc} \frac{\partial G_{\infty }}{\partial z}=\frac{1}{\sqrt{1-x^{2}}}, & \text{on}|x|\leq 1,z=0 \end{array}$$

the first integral on the right-hand side of Equation (79) can be evaluated to be:

(81)
$$2\int_{1}^{1-\delta }{\left(\frac{\partial G_{\infty }}{\partial z}\right)}^{2}\text{d}x=\log2-\log\delta +O(\delta )$$

Substituting *x* = 1 − *β*^−1^*u* into the second integral on the right-hand side of Equation (79), we obtain:

(82)
$$2\int_{1-\delta }^{1}\frac{\partial G}{\partial z}\frac{\partial G_{\infty }}{\partial z}\text{d}x\sim \frac{2}{\sqrt{\beta }}\int_{0}^{\delta \beta }\frac{\partial G}{\partial z}\frac{1}{\sqrt{2u}}\text{d}u$$

where *δβ* ≫ 1. But as *u* → ∞, *G* ∼ *G*_∞_, so that for *u* > *M* for some large constant *M*:

(83)
$$\frac{\partial G}{\partial z}\sim \frac{\partial G_{\infty }}{\partial z}\sim \frac{\sqrt{\beta }}{\sqrt{2u}}$$

Therefore

(84)
$$\frac{2}{\sqrt{\beta }}\int_{0}^{\delta \beta }\frac{\partial G}{\partial z}\frac{1}{\sqrt{2u}}\text{d}u\sim \int_{M}^{\delta \beta }\frac{1}{u}\text{d}u+\frac{2}{\sqrt{\beta }}\int_{0}^{M}\frac{\partial G}{\partial z}\frac{1}{\sqrt{2u}}\text{d}u$$

The contribution from the first integral on the right-hand side dominates, so that:

(85)
$$2\int_{1-\delta }^{1}\frac{\partial G}{\partial z}\frac{\partial G_{\infty }}{\partial z}\text{d}x\sim \log\delta +\log\beta$$

Adding the two contributions from Equations (81) and (85), we find that:

(86)
$$\frac{1}{\pi \beta }\int_{-1}^{1}\frac{\partial G}{\partial z}\frac{\partial G_{\infty }}{\partial z}\text{d}x\sim \frac{1}{\pi \beta }+\log\beta ,$$

and hence, from Equation (78), that:

(87)
$$Q(\beta )\sim \log2+\frac{1}{\pi \beta }\log\beta$$

## Supplementary Files

Supplementary File 1 Q_beta_working_curve.csv (CSV, 53 KB)
